# Expression of the *odd-2* Gene in *C. elegans*

**DOI:** 10.17912/micropub.biology.000967

**Published:** 2023-08-25

**Authors:** Elizabeth Charnysh, Keith R. Strohmaier, Joel H. Rothman, Amy C. Groth

**Affiliations:** 1 Biology Department, Eastern Connecticut State University, Willimantic, CT, USA; 2 Department of MCD Biology and Neuroscience Research Institute, University of California Santa Barbara, Santa Barbara, CA, USA

## Abstract

The *odd-2* gene in *C. elegans* is an orthologue of the *odd-skipped* gene in *Drosophila* and the odd-skipped related genes in mammals. The mammalian genes have been shown to be expressed in a variety of tissues and cancers. It was previously reported that ODD-2 is expressed in the intestine and shows some expression outside of the intestine in the tail region. Using a partial ODD-2 ::GFP fusion, we hypothesize that the expression outside of the intestine may be in rectal gland cells, and we also report that ODD-2 may be expressed in the germline sheath cells.

**Figure 1. Expression of ODD-2 ::GFP in the intestine, the tail region and sheath cells:**
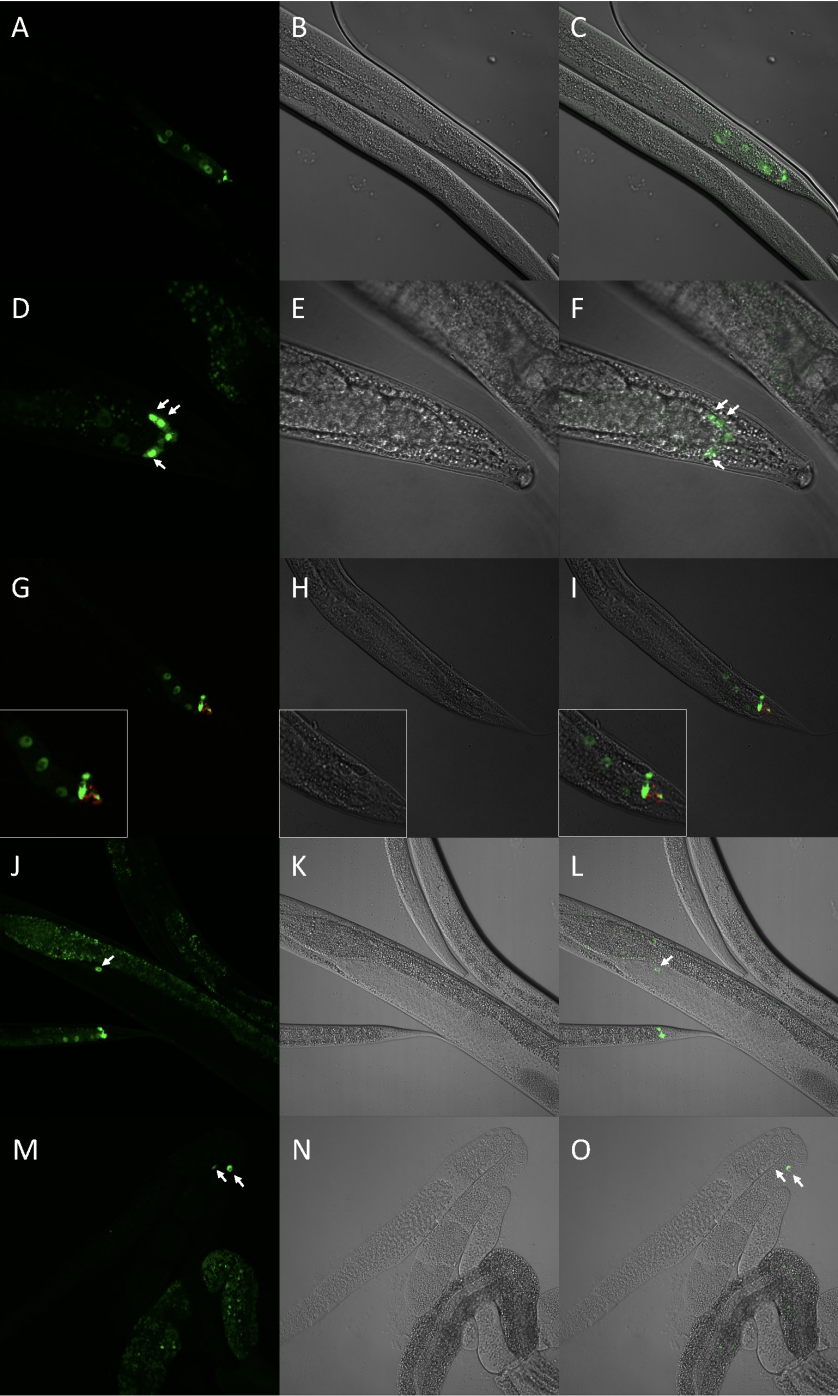
Images were taken of JR2005 or ACG10 with an Olympus BX61 confocal microscope. All images show a single plane of view, except D and F which depict the GFP as a Z projection. In each set of three images, the left image is the fluorescent image, the middle is the brightfield, and the right image is the merge. Worms were immobilized with levamisole; in D-F, they were immobilized with levamisole and Nemametrix Nemagel. A-C) Images of a JR2005 worm at 40x magnification showing expression in the posterior intestinal cells and cells posterior to the intestine. D-F) Images of a JR2005 worm at 100x magnification. Possible rectal gland expression is indicated by white arrows. The GFP is a Max Intensity Z projection of 11 slices. The brightfield image is the middle slice. G-I) Images of an ACG10 worm at 40x magnification showing that BUS-1 expression (red) is largely posterior to the ODD-2 expression (green). Insets show enlarged versions of the end of the tail region. J-L) Images taken at 40x show expression of ODD-2 in the vicinity of the germline. Arrows indicate the cell outside of the intestine that is expressing ODD-2 (likely a sheath cell). M-O) Images show an extruded germline at 40x magnification with two likely sheath cells expressing ODD-2 indicated by arrows.

## Description

The transcription factor genes *odd-1* and *odd-2* in *C. elegans* are homologs of the *Drosophila* *odd-skipped* family of genes and the mammalian Odd-skipped related 1 and 2 ( *OSR1* and *OSR2* ) genes (Buckley et al., 2004) . *Odd-skipped* ( *odd* ) was named because every odd body segment is skipped when the *odd* gene is deleted in *Drosophila* (Coulter et al., 1990) . The mammalian *OSR* genes have been implicated in the development of a number of tissues and a variety of cancers (Tena et al., 2005; Wang et al., 2007; Wang et al., 2018; Yu et al., 2022) . The C-terminal DNA-binding domain of ODD-2 is more closely related to the mammalian proteins, while the DNA-binding domain of ODD-1 is more distantly related. Both *C. elegans* genes encode three zinc-finger DNA binding domains, as does the mammalian *OSR1* , while the mammalian *OSR2* has five (Buckley et al., 2004) . According to the GTex database (gtexportal.org), *OSR1* is expressed at the highest levels in arteries (aorta is the highest with 154.1 transcripts per million (TPM)), bladder, visceral fat, esophagus, salivary gland, breast and colon (32.56 TPM) and is present at fairly low levels in most tissues, but not expressed in the brain. *OSR2* is expressed most highly (in decreasing order) in the uterus (254.3 TPM), fallopian tube, cervix, ovary and vagina (85.19 TPM), followed by tibial nerve (TPM 55.12), testis, prostate, subcutaneous fat and bladder (TPM 30.88). There is lower expression in most tissues, and no expression in the brain.

The expression pattern of an *odd-2 ::gfp* reporter in embryos and larvae has been previously reported (Buckley et al., 2004) . Early larvae showed ODD-2 expression in the anterior and posterior intestine, as well as in unidentified posterior cells outside of the intestine. The strain used in that study was unstable, preventing further studies of expression (Buckley et al., 2004) . Here, we describe the expression of an *odd-2 ::gfp* reporter in adult worms.

Expression of the partial fusion strain JR2005 ( ODD-2 ::GFP) was studied in adult worms by fluorescence microscopy. GFP showed bright expression in the adult posterior intestinal cells and also very strong expression in a cluster of three to four cells posterior to the intestine. Morphology indicated that these cells possibly included the three rectal gland cells ( Figure 1A -F). Rectal gland expression is supported by single cell transcript analysis (Packer et al., 2019) . Additionally, we often saw one or two green cells in or near the bend in the anterior and/or posterior germlines ( Figure 1J -L). We observed at least one of these germline-localized cells in ~70% of L4-adult worms, although it is possible that expression was sometimes obscured by the intestine and that this number is higher. These cells looked similar in appearance to the expression in the posterior intestinal cells, but were localized outside of the intestine. Based on their position and appearance, we hypothesize that the expression is in sheath cell nuclei. (It should be noted that conclusions about subcellular localization of ODD-2 cannot be drawn, since the construct is a partial fusion and may not contain the appropriate signal sequences.) Although one cell was often bright in appearance, these cells were generally dimmer than the intestinal and posterior extra-intestinal cells.

In order to further explore the expression in the tail region, we crossed the *odd-2 ::gfp* reporter to a *bus-1 ::dsRed2* reporter (present in strain CB6372 ) to create the strain ACG10 . BUS-1 has previously been shown to localize to the rectal epithelial cells (positioned directly behind the rectal gland cells), as well as some tail neurons and two head neurons (Gravato-Nobre and Hodgkin 2008) . The penetrance of the *bus-1* reporter is low, and we were not able to generate a stable line. However, we were able to observe a few worms and found that, as expected, the *bus-1* reporter was expressed in cells that were immediately posterior to the *odd-2* reporter expression with no colocalization of the two signals ( Figure 1G -I). This finding supports our hypothesis that ODD-2 is expressed in the rectal gland cells, but it is not definitive. Also, as seen in Panels A, C, D, F, G and I, there is a focus of expression posterior to the three cells around the outside of the intestine – this may be expression in projections of those same cells, or potentially an additional cell or cells. Further colocalization studies are needed to draw more definitive conclusions.

To support our hypothesis that ODD-2 is expressed in the sheath cells, we extruded germlines from adult worms. Expression appeared to be on the edge of the germlines, generally near the bend in the gonad ( Figure 1M -O). This study is the first report of expression of *odd-skipped* genes in *C. elegans* outside of the alimentary system. As the human OSR2 is expressed in many tissues related to the female reproductive system, exploration of a possible role of ODD-2 in the worm reproductive system warrants further study. Indeed, recent publications have indicated that the down-regulation of OSR2 plays a role in human infertility (Ma et al., 2022) and that OSR1 acts as a tumor suppressor in ovarian cancer (Yu et al., 2022) .

We note that the strain used in this study is an unoutcrossed, integrated, multi-copy array and encodes only the first 55 amino acids of the ODD-2 protein in the fusion, so the findings reported here are tentative. Some ectopic expression attributed to plasmid sequences has been reported in the posterior intestine (Krause et al., 1994) so it is conceivable that the intestinal expression is an artifact, although it is consistent with other studies (Buckley et al., 2004; Packer et al., 2019) .

## Methods

*Strains and Reagents*

JR2005 [ *wls123* ] was constructed by integrating an array, pEX1234, which contained pRF4 [ *rol-6 (gf)* ] and a construct containing ~2.5 kb of upstream *odd-2* sequence and part of the first exon (encoding the first 55 amino acids) of the *odd-2* gene fused to lacZ GFP. The fragment was amplified from cosmid C34H3 using primers KRS56 (AACACGAAGCTTCCCAACAT) and KRS57 (TGGGATCCGTGATCATTGGA) and cloned into vector pCR2.1. The fragment was then subcloned into vector pPD96.04 using the restriction enzymes BamH1 and HindIII. The length of the fragment excluding restriction sites is 2529 bp. The array was integrated by UV and has not been outcrossed. The junction was verified by PCR and sequencing using the primers ACG380 (ATGCTTCCGTGGCAACGACAAG) and ACG439 (AAAGGGCAGATTGTGTGGAC). CB6372 , a gift of the Hodgkin lab, contains *eEx557* , a *bus-1 ::dsRed2* reporter (genotype: *eEx557 [ rol-6 (gf); bus-1 ::dsRed2]* ).

*Imaging*

Worms were immobilized in 0.25 mM levamisole or levamisole plus Nemametrix Nemagel (InVivo Biosystems, Eugene, OR) and imaged on an Olympus BX61 confocal using the 40x or 100x objective. Germlines were extruded by placing the worms in M9 containing 0.1% Tween and 0.25 mM levamisole and removing their heads with a scalpel.

## Reagents

**Table:** 

Strain	Genotype	Source
JR2005	*wIs123 [ rol-6 (gf)* ; *odd-2 ::lacZ GFP* ]	Joel Rothman
N2	Wildtype	CGC
CB6372	*eEx557 [ rol-6 (gf* ) *; bus-1 ::dsRed2* ]	Jonathan Hodgkin
ACG10	*wIs123 [ rol-6 (gf); odd-2 ::lacZ GFP]* ; *eEx557 [ rol-6 (gf); bus-1 ::dsRed2] *	Amy Groth (strain was unstable and unable to be maintained)
